# Postnatal experiences of women with cardiac conditions: a systematic review and meta-synthesis

**DOI:** 10.1016/j.xagr.2025.100564

**Published:** 2025-09-01

**Authors:** Jasmine X. Kiley, Annabelle Corlett, Emma Mitchell-Sparke, Brittany Jasper, Tabitha Wishlade, Catriona Bhagra, Sara Wetzler, Catherine E. Aiken

**Affiliations:** 1 Department of Obstetrics and Gynaecology, University of Cambridge, The Rosie Hospital and NIHR Cambridge Biomedical Research Centre, Cambridge, UK (Kiley, Mitchell-Sparke, Jasper, Wishlade, and Aiken); 2 Department of Clinical Medicine, University of Cambridge, Addenbrooke’s Hospital, Cambridge, UK (Corlett); 3 Tufts University School of Medicine, Boston, MA (Mitchell-Sparke); 4 Department of Cardiology, Addenbrooke’s and Royal Papworth Hospitals, Cambridge, UK (Bhagra); 5 Department of History and Philosophy of Science, University of Cambridge, Free School Lane, Cambridge, UK (Wetzler); 6 Department of Obstetrics, Gynecology, and Reproductive Science, Icahn School of Medicine at Mount Sinai, New York, NY (Wetzler)

**Keywords:** cardiac condition, cardiac disease, high-risk pregnancy, patient experience, postnatal, postpartum, qualitative study, systematic review

## Abstract

**Objective:**

The leading cause of maternal mortality in high-income countries is cardiovascular conditions. The highest risk period for women with cardiac conditions is usually the early postnatal phase; however, postnatal care is often under-resourced. We aim to inform supportive care pathways that address the medical and emotional needs of women with cardiac disease during the postnatal period.

**Data Sources:**

To identify studies, Medline via Ovid, Embase via Ovid, CINAHL via EBSCO, PsycINFO via EBSCO, Scopus, Web of Science Core Collection, and ASSIA via ProQuest were searched (database inception—June 2025).

**Study Eligibility Criteria:**

Studies reporting qualitative data about the postnatal experiences of women with any cardiac condition globally were included. The postnatal period was defined as the events occurring between delivery and 1 year postpartum.

**Study Appraisal and Synthesis Methods:**

The Critical Appraisal Skills Programme checklist for qualitative research was used to perform quality assessment and reduce risk of bias. Inductive coding and thematic analysis were performed using NVivo v.15.

**Results:**

Eleven qualitative studies were included in the meta-synthesis. We identified a chronological framework to describe the postnatal experiences of women with cardiac conditions. We identified key themes of (1) initial fragmentation of attention, (2) physical and psychosocial transitions, and (3) planning for the future. Women described a fragmentation of attention after delivery of their infant, as their own mindset frequently shifted toward motherhood, while their care team focused more on the infant and maternal cardiac recovery. Their experience of motherhood often differed from their expectations, adding to feelings of stress around their postnatal experience. Emotional recovery from complex pregnancies was influenced by desire for future pregnancies and by the degree of support available from family and wider community.

**Conclusion:**

Cardiac conditions profoundly influence postnatal emotional and psychosocial well-being. During the early postnatal phase, care must balance the needs of mother, infant, and maternal cardiac condition. Postnatal cardio-obstetric care should include debriefing appointments with providers, with thorough, sensitive discussion of the risks of potential future pregnancies. Supporting women’s transition into motherhood is critical to help them process experiences and engage with care to improve their long-term cardiac prognosis.

**AJOG Global Reports at a Glance**

**Why was this study conducted?**

This study was conducted to construct a chronological framework informing supportive postnatal care for women with cardiac conditions.

**Key findings**

Care during the early postnatal phase must sufficiently balance the needs of mother, infant, and care relating to their cardiac condition. Sensitive and in-depth communication is important in helping women understand and navigate their cardiac prognosis, particularly regarding the risks of future pregnancies.

**What does this add to what is known?**

Our framework may improve understanding of the postnatal experiences of women with cardiac conditions by providing context as to how cardiac conditions impact emotional and psychosocial well-being. Providing sensitive communication and support during the postnatal period may help address the disrupted transition to motherhood and conflicting emotions experienced by many women with cardiac conditions.

## Introduction

Cardiovascular conditions are currently the leading indirect cause of maternal death in high income settings.^1^ In the United Kingdom, maternal mortality rates due to cardiac conditions increased between 2020 and 2022, despite decreases in the three preceding triennia.^2^ Cardiovascular conditions have also been identified as major drivers of the rise in maternal mortality in the United States in recent years.^3^ As the average age of the maternity population rises and comorbidities such as maternal obesity and diabetes become more common, the prevalence of maternal cardiovascular conditions is likely to continue to increase.

The highest risk of death for women who experience pregnancy with a cardiac condition occurs in the postnatal period.^4^ Following gradual adaptations within the cardiovascular system to the demands of pregnancy over ∼37 weeks, the days following delivery present a challenging period of abrupt shifts in circulating blood volume, extracellular volume, blood pressure, and oxygen-carrying capacity.^5^^,^^6^ These postpartum cardiovascular challenges may be further exacerbated by concomitant stressors, including postpartum hemorrhage or infection. Paradoxically, postnatal care is often perceived as the least resourced and least vigilant phase of maternity care. In the UK, the NHS maternity survey (2024) showed postnatal women were more likely to report that their care needs were poorly met than at any other point in the maternity timeline.^7^ Moreover, for many women, the early postpartum period can be a highly stressful experience with profound mood and hormonal shifts, along with changing healthcare priorities as they transition to motherhood. There are additional challenges for women with cardiac conditions, as they experience increased risks of delivering a premature baby, recommendations for medications incompatible with breastfeeding, and higher rates of complex deliveries. The synergistic impact of high cardiac risk, reduced vigilance in care, and multiple psychological stressors makes the postnatal period a critical time window in the medical management of women with cardiac conditions. It is essential to understand how women with cardiac conditions experience the postnatal period, and thus to better understand how they can best be supported throughout postpartum recovery.

Antenatal experiences of women with cardiac conditions have been explored in existing literature,^8^^,^^9^ but little has been reported specifically about postnatal experiences. In order to establish pathways that provide safe and comprehensive care during this intense and risky transition period, we aim to understand the reported experiences and expectations of women with cardiac conditions in the postnatal phase. We performed a qualitative systematic review investigating the postnatal experience of women with cardiac conditions with the goal of informing safe and supportive care practices.

## Methods

### Search strategy

The review protocol was prospectively registered (PROSPERO: CRD42025635377) and results are reported in line with PRISMA guidelines. To identify studies, seven databases were searched (Medline via Ovid, Embase via Ovid, CINAHL via Ebsco, PsycINFO via EBsco, Scopus, Web of Science Core Collection, and ASSIA via ProQuest). The initial search identified articles pertaining to cardiac conditions and pregnancy, followed by filtering for articles specifically reporting postnatal experiences (A.1). We also conducted a manual search of the reference lists of included studies, Google Scholar, and previous systematic reviews to achieve a comprehensive search. All searches were completed on June 24, 2025.

### Selection criteria

All original studies reporting qualitative data (mixed methods or qualitative design) in peer-reviewed journals that met the eligibility criteria were included. The participant population consisted of any pregnant woman with a cardiac condition. We defined the postnatal period for the purpose of this study as reflections on the events that occurred between delivery and 1 year postpartum. Studies that did not report women’s postnatal experiences, studies in languages other than English, and papers other than primary research publications (eg, review articles) were not selected. After deduplication, all articles were independently screened by at least two reviewers (JK, AC, or BJ) using Rayyan software and blinded to each other’s decisions.^10^ A third reviewer (CA) was available to resolve differences of opinion. Full texts of all articles that met selection criteria were obtained.

### Assessment of risk of bias

The studies in this review were evaluated using the Critical Appraisal Skills Programme (CASP) 18 toolkit. Two reviewers (JK and EM) conducted independent assessments, and a third reviewer (CA) was available to resolve conflicts.

### Data synthesis

Data was extracted from included studies using NVivo v.15 software to code first-order (participant quotations) data. Second-order data (researcher interpretations) was employed to understand the context of the data and inform the coding process. Two reviewers (JK and AC) corroborated the extracted data, and conflicts were resolved in discussion with a third reviewer (CA).

Two authors (JK and CA) completed the inductive thematic analysis to formulate descriptive analytical themes and devise a chronological framework to outline the postnatal experience of women who experienced a pregnancy with a cardiac condition.

## Results

### Study selection

Our search yielded 18,065 studies, with 10,042 unique studies screened after removal of duplicates (A.1). We included 11 studies of 364 pregnancies in the final thematic synthesis (Table).

**Table:** Summary of included primary study characteristics

Study	Country	Diagnosis and sample size	Study focus	Qualitative prompts	Data collection	Data analysis	Summary of themes	No. of references
de Wolff et al^16^	Denmark	Peripartum cardiomyopathy (*n*=24)	Experiences of psychological adaptation after having PPCM	Opening question and probe examples provided	Interviews	Thematic analysis	Recovering to a new normal after PPCM, losing trust, silence after chaos, disruption of early mothering, choices made for me and not by me, ability to mobilize inner resources	47
Dekker et al^18^	International online support groups (My Heart Sisters, A Woman’s Heart, PPCMnet)	Peripartum cardiomyopathy (*n*=92)	Experiences of women after being diagnosed with PPCM	N/A	Narratives from online support groups	Thematic analysis	Symptom dismissal and misdiagnosis, strong emotional reactions upon learning of diagnosis, concerns about impact of PPCM on future childbearing	19
Donnenwirth and Hess^11^	United States	Peripartum cardiomyopathy (*n*=16)	Explore experiences of women living with PPCM and their decisions regarding a subsequent pregnancy (SSP)	Open-ended sample questions provided	Interviews	Constant comparison analysis	Risk of relapse into heart failure impact decisions about future pregnancies, receiving the ultimatum “no more children,” weighing the risks of a SSP, making the decision about a SSP, experiencing a SSP	31
Hess and Weinland^21^	International online support group (MySpace)	Peripartum cardiomyopathy (*n*=148)	Describe contents of postings made on MySpace by women diagnosed with PPCM	N/A	Narratives from online support groups	Thematic analysis	Discussion of symptomology, exchange of advice, interactions with healthcare providers, uncertainty of future pregnancies, expressions of spirituality, recovery from heart failure	21
Hess et al^20^	United States	Peripartum cardiomyopathy (*n*=12)	Determine benefits of participation in the online support group for PPCM	Open-ended and Likert-style questions provided	Descriptive survey	Thematic analysis	Benefits of being in group, how support group helps me, what I learned from others in group, what I gain as part of group	38
Hutchens et al^12^	Australia	Tetralogy of Fallot (*n*=2), bicuspid aortic valve (*n*=1), mitral valve prolapse (*n*=1), hypertrophic cardiomyopathy (*n*=5), arrhythmogenic right ventricular dysplasia (*n*=1), long QT syndrome (*n*=2), bicuspid aortic valve and patent ductus arteriosus (*n*=1), left ventricular noncompaction cardiomyopathy (*n*=1), idiopathic cardiomyopathy (*n*=1), pregnancy-associated spontaneous coronary artery dissection (*n*=10), patent foramen ovale (*n*=1), peripartum cardiomyopathy (*n*=1)	Explore and understand the healthcare experiences of women with cardiac disease in pregnancy and postpartum	Interview guide not specified	Interviews	Inductive reflexive thematic analysis	Dismissed: struggling to be heard; too little, too unclear: in search of information; winging it: research, education, and guidelines; fragments: care co-ordination and continuity; making do: fitting into services designed for others	42
Hutchens et al^14^	Australia	Congenital heart disease (*n*=4), genetic heart disease (*n*=8), acquired heart disease (*n*=12), congenital and genetic heart disease (*n*=1)	Correct the lack of visibility and information on the experiences of women with cardiac disease in pregnancy and the first year postpartum	Opening question provided, interview guide not specified	Interviews	Interpretive inductive thematic analysis	Ground zero: index events and their emotional and psychological impact; self-perception, identity and worthiness; one the road alone: isolation and connection	63
Liu et al^17^	Taiwan	Congenital heart disease (*n*=11)	Explore the essence of lived experience of first-time mothers with CHD	Interview questions provided	Interviews	Empirical phenomenological analysis	Recognizing pregnancy risks, performing self-care for health, building self-worth from my baby, adapting to postpartum life and adjusting priorities, enjoying being a first-time mother, factors contributing to success in high-risk childbirth	35
Mayer et al^13^	United Kingdom	Cardiac disease (arrhythmic or structural) (*n*=15)	Describe composition and processes of multidisciplinary care between maternity and cardiac services and women’s experiences of delivering/receiving care within these models	Interview topic guide specified	Retrospective case note audit (*n*=42 women), interviews (*n*=15 women, *n*=11 clinicians)	Thematic analysis	Model of integrated care, influence of clinicians’ specialist interest in pregnancy and cardiac conditions on model of care, clarity of acquired vs congenital pathways, congenital conditions, acquired conditions, midwifery involvement, normality	51
Patel et al^15^	Sweden	Peripartum cardiomyopathy (*n*=19)	Explore and describe women’s experiences of symptoms in peripartum cardiomyopathy	Opening question and probe examples provided	Interviews	Qualitative content analysis	Being caught in a spider web, invasion of the body by experienced symptoms and feeling of helplessness	41
Patel et al^19^	Sweden	Peripartum cardiomyopathy (*n*=19)	Explore women’s experiences of health care while being diagnosed with PPCM	Open-ended and probe sample questions provided	Interviews	Qualitative content analysis	Exacerbated suffering, not being cared about, not being cared for, not feeling secure	40

### Study characteristics

Studies were conducted in six countries (Australia, Denmark, Taiwan, United Kingdom, USA, Sweden) and using data accrued from internationally accessible online support groups (OSGs). At least 13 distinct cardiac diagnoses were represented among the participants (Table). The research methods of the studies included interviews (*n*=8), surveys (*n*=1), and narratives from OSGs (*n*=2). The analytic methods employed in the included studies were thematic analysis, constant comparison analysis, empirical phenomenological analysis, and qualitative content analysis.

### Risk of bias of included studies

The overall quality of included papers was good, as assessed by the CASP toolkit. The studies met the CASP 18 evaluation on the majority of key points (A.2). Two studies had unclear recruitment strategies, and four contained ambiguity in the relationship between researcher and participants. There was one study where data analysis strategy was unclear.

### Synthesis of results

We identified the delivery of the baby as a watershed moment in a woman’s experience as a cardio-obstetric patient. Women experiencing pregnancy with a cardiac condition are often subject to intensive antenatal monitoring, but as soon as birth occurs, the care team’s focus shifts to accommodate both mother and baby as distinct entities. In the minutes, days, and months following birth, women perceived themselves as transitioning to motherhood. We found that this mindset shift could sometimes overshadow the previous focus on their cardiac health for both women and their care providers.

**Figure 1:**
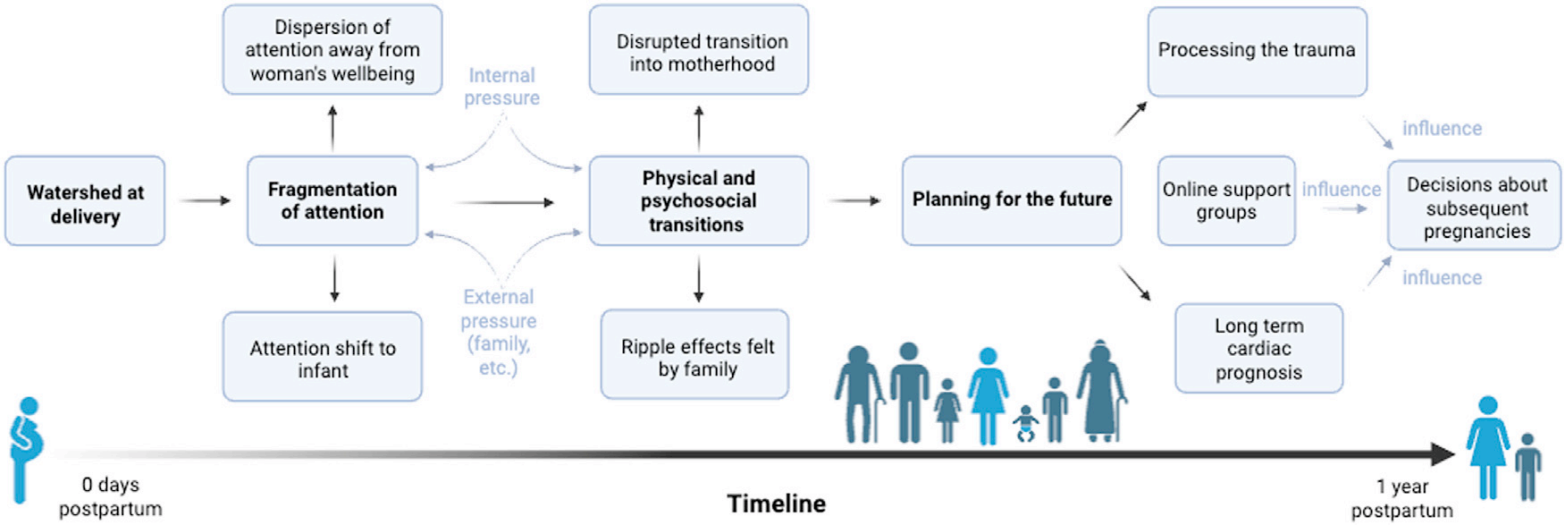
Chronological framework for postnatal experiences of women with a cardiac condition

We determined three key themes that form a chronological framework for the experiences of women with a cardiac condition over the year following the birth of their baby: (1) initial fragmentation of attention, (2) physical and psychosocial transitions, and (3) planning for the future (Figure 1).

Kiley. Postnatal experiences of women with cardiac conditions. AJOG Glob Rep 2025.

We focus primarily on the difficulties faced by many women with cardiac conditions after pregnancy, as these represent key opportunities for improving maternity care. However, it is important to note that women also spoke of how positive and empowering their experiences with pregnancy and birth had been.

*My blood pressure stayed good the whole pregnancy. My ejection fraction stayed 50-55% throughout the pregnancy as well. I am not on any medication. I feel great. I love being a mom. Being a mom is one of the best roles that I have ever had in my life. I feel that coming through […] without any relapse, I feel like I ran a marathon and I won. I feel that I am very lucky.*^11^ (USA)

#### Subtheme 1: Initial fragmentation of attention

Up until the point of delivery, mother and baby often exist as a singular entity in the view of both women and their providers. Maternal health largely dictates that of the fetus in cardio-obstetric care prior to birth, and the care team thus tend to focus on them as a unit. At birth, women reported a sense of this entity splitting and the subsequent attention of the care team splintering, with the mother’s health and well-being sometimes being relegated to one of many considerations.

##### Dispersion of attention away from aspects of the woman’s well-being

Many women felt that the attention paid to their cardiac condition was insufficient in the immediate postpartum period. Often their care was provided solely by obstetricians and midwives, who were perceived as ill-equipped to provide treatment or medical advice for their cardiac condition. Many women reported feeling that their physical condition was no longer treated as carefully.

*Oh, no, no. Just go down there [to the NICU] and if you feel like you’re going to pass out, sit down on the floor and just yell out and someone will hear you.*^12^ (Australia)

Beyond the immediate postpartum period, many women had ongoing concerns that their cardiac condition was not well-monitored or sufficiently integrated into their postnatal care.

*I saw the cardiologist six months after I delivered, nothing before the six months, I haven’t seen any one or spoken to anyone about how I feel or what happened … the midwife visits I had but no one asked about my heart.*^13^ (United Kingdom)

However, other women experienced care that was highly focused on their physical well-being. Instead, they discussed a lack of attention to their mental and emotional recovery and often expressed feeling unsupported in their transition to motherhood.

*I was in a shared room with older men with only a curtain between us, I’m having to sit there breast pumping, I’ve got my newborn in there … it was a pretty horrendous experience, the whole thing.*^14^ (Australia)

*So, physical care is one thing, but I think anything around mental health in it is just critically important. Because that’s the thing that’s either going to get you through the rest of your life or not, or hold you in a space that you just won’t be able to move from … this experience for me wasn’t very long ago, and I don’t feel I was offered nearly enough assistance around that.*^12^ (Australia)

*…as I started to get a little bit physically better was probably when … mentally and emotionally things started to fall apart for me.*^12^ (Australia)

In common with women who felt under-monitored for their heart condition, women who felt over-monitored recognized that this extended beyond the immediate postpartum period. Many women discussed their cardiac monitoring overshadowing their mental and emotional well-being, including intruding on their ability to settle into new routines of family life.

*I was referred to a special clinic … I had to go there every day for check-ups after leaving two other kids in kindergarten and it was very strenuous.*^15^ (Sweden)

Women often described how the shift in attention away from their overall well-being left them feeling isolated. In contrast with their pregnancy experiences, during their postnatal recovery, they felt less well-understood by those around them.

*“Oh, my God, your husband’s so amazing. He’s doing these great things with the kids.” I’m like, “Well, yes, I get it, but I nearly died here and I’ve got no choice and what about me hey?”*^12^ (Australia)

##### Attention shift toward their infant

When attention shifted away from the mother’s well-being, it was often redirected toward their infant. Women recounted experiences of their own healthcare not being the priority of their providers, who they felt were more concerned about the welfare of the newborn.

*I had to nag and fuss for pain medication … it hurt to breathe, but they said “you just want painkillers and to avoid breastfeeding.” I was very sad and felt terribly bad, felt like a drug addict.*^15^ (Sweden)

For other women, a shift in focus toward the newborn felt entirely appropriate, which began at birth and continued later the postpartum recovery process. Some women also experienced a profound change in their own health concerns: for example, transferring from worrying about themselves to their baby.

*…I’ve been focusing on my boy and I’ve focused on him and him and only him, so when I was sure that he was fine, then to hell with me …. I mean, I’ve always been like that: my children come first and then we could see if there was any time left for me, right?*^16^ (Denmark)

*Because both of going to work and taking care of my baby are so tiring, arrhythmia becomes the last consideration.*^17^ (Taiwan)

#### Subtheme 2: Physical and psychosocial transitions

##### Disrupted transition into motherhood

Many women experienced disappointment and frustration during their transition into maternal life. Especially when their expectations of early motherhood were not fulfilled, this was an intensely stressful experience. Many resented the intrusion caused by monitoring and treating their cardiac condition during a time they felt should have been focused on enjoying their new infant.

*And then the other part of it was … that process of working through what had happened. I was going to the mothers’ group … I remember I felt like I’d been somewhat cheated. The others sat there, and it was so fantastic, the early stages were a fantastic time… and I remember I felt I wasn’t part of it, and it wasn’t their fault, it was just me, I remember I really felt cheated on. Rather a strange word to use, but I can’t find a better way to describe it, I felt like I’d been robbed of something.*^16^ (Denmark)

As women began to process their pregnancy and birth experience, many developed significant postnatal anxiety. Particularly in the postnatal phase after leaving hospital, women described trauma responses connected with their experiences of pregnancy with a cardiac condition. For some women, their health anxiety was contextualized by potential impacts on their infant.

*I couldn’t handle going shopping. I felt anxious about… I mean, I just felt that the items were falling on my head. I was afraid of being home alone. I was afraid of sleeping at home on my own. My husband travels a lot, so he was away a few times. I had to have my mother, or my friends stay overnight.*^16^ (Denmark)

*I was scared out of my wits, and I got to the stage where I couldn’t push the pram for fear that if I had [another] heart attack, the pram would be pushed under traffic or something. I wasn’t only fearful for myself, it was more for the baby.*^12^ (Australia)

##### Infant feeding

Choice, and lack of choice, around feeding their babies were frequently discussed by women. For some, holding onto their choice to breastfeed despite the impact of their cardiac condition was an important part of maintaining control, not only over their transition to motherhood, but also their bodies.

*I remember that I had breastfed. And for me that was an important thing. I could tell that it had been important for our elder daughter to be breastfed. She looked me straight in the eye and took my hand. There was so much contact… So that’s when I decided that it was something I was really going to fight for (breastfeeding).*^16^ (Denmark)

Many women with cardiac conditions were advised not to breastfeed, either because of concern regarding the impact of medications present in breastmilk on their babies or because providers felt that the hormonal impact of breastfeeding might be detrimental to their cardiac condition. Where this was advised, women often reported feelings of frustration and sadness, and for some, this altered their view of themselves as mothers.

*It had been a bit hard to begin with (breastfeeding), so it was lovely when it started going well, and it was easy too. And I felt I had to throw that away. And then at that point I thought it was annoying that I wasn’t allowed to (carry on breastfeeding), because I really felt like failure.*^16^ (Denmark)

*I was fortunate to have a sister who had a baby just 5 weeks before me who was able to pump a bottle of breast milk for my baby everyday, but that same act of love also depressed me and was a constant reminder that I wasn’t well.*^18^ (OSG)

By contrast, other women reported very positive experiences with infant feeding despite their cardiac condition, and that this was a key part of family bonding.

*It wasn’t something I missed doing (breastfeeding). Because we have had so much joy with them bottle-feeding …. and the four months when my husband was home …it was nice. We sat there in the night with a baby each, you know?*^16^ (Denmark)

##### Ripple effects on other family members

Many women reported that their families also experienced the repercussions of their cardiac condition in the postnatal period. This was often experienced as yet another layer of burden within the newfound responsibilities of motherhood that women were learning to juggle.

*[Cardiomyopathy] stole so much from me and my family. My sister almost didn’t get pregnant because of what she saw me go through. She only had one child and had so much anxiety through her pregnancy that she didn’t have another. That makes me feel guilty. Sometimes I look at the ripple effect that [cardiomyopathy] caused and I want to take a sword to it and cut it in half …. like it did me.*^18^ (OSG)

*I think it is only years later that the toll has become very evident, for the whole family, in terms of mental health. I think the whole family has struggled with it … That’s the fallout really.*^12^ (Australia)

#### Subtheme 3: Planning for the future

##### Processing the trauma

Women described a striking impact of their pregnancy and birth experiences on their psychological health, and many described a lengthy period of trying to process conflicting emotions following birth.

*I’m glad that I lived to see [my daughter’s] birthday. I’m bitter. I’m thankful. I’m grateful to be alive. I’m pissed. I’m all of these things.*^18^ (OSG)

The experience of psychological recovery from a pregnancy and birth with a cardiac condition was difficult for many women. There was a strong sense that mental health recovery was more difficult and less supported than physical recovery.

*I have really struggled with it—it’s been a year and I’m still kind of, you know, grappling with all that sort of stuff.*^12^ (Australia)

*Although I don’t feel sick all the time, a constant reminder is there, making me anxious and sad….*^19^ (Sweden)

*…[Although] my heart’s healed; I still have the psychological healing to do.*^12^ (Australia)

During the months following birth, women with cardiac conditions who had experienced trauma described utilizing various strategies to process their experiences. Many women reported a strong sense of striving to regain a sense of control. Women often referenced their own determination in attaining this, whereas others described leaning on external resources.

*It’s simply my own willpower. I want to go out walking. At the start I couldn’t walk… I couldn’t push a pram… I mean, I had pain in my chest. But in the end, I was able to go for long walks.*^16^ (Denmark)

*I really needed to know that there were others out there who were in the same situation. I needed to know that it was OK to be scared and confused. [The women in the online group] gave me hope that it wasn’t just a death sentence like the doctors had made it out to be.*^20^ (OSG)

*So, she (the psychologist) gave me some easy and simple methods or solutions for getting through these anxiety attacks I had, and that was great. That was what I really needed.*^16^ (Denmark)

Other women reflected that they had experienced a lasting shift in their perspective or identity in the wake of their pregnancy complicated by a cardiac condition. This transition was experienced by women in different ways, with some reframing the change as positive and others as something entirely outside of their control.

*When you’re diagnosed with something like this that you’ve always had, you’re sort of left feeling a bit empty and weird. You have to just keep going now, but you’re a different person … everything’s different.*^12^ (Australia)

*People in the older generation think that giving birth to an abnormal child means that the mother did something wrong in her previous life. Therefore, this is like a sort of vengeance for me.*^17^ (Taiwan)

*But it has made us look differently at what are problems and what are just petty things. We put things in perspective. So, we’ve tried to see whether we could get something positive out of it instead. Because we couldn’t do anything about it, I mean… So, we’ve tried to use it… I mean, for something good….*^16^ (Denmark)

*It really makes you just see that your life can be altered at any moment and that you can be really at the will of it—of whatever it is that God wants.*^11^ (USA)

##### Decisions about subsequent pregnancies

Hearing from their provider that a subsequent pregnancy was ill-advised was a pivotal and sometimes devastating experience for many women. Their instinct that their family was not complete was difficult to reconcile with the danger posed by their cardiac condition. Women talked about the conflicting emotions they faced in deciding whether they would embark on subsequent pregnancies.

*I am finding it hard to deal with the fact that I am told not to have any more children. I long to experience the miracle of birth again….*^21^ (OSG)

*I mean, I think I’m missing one (a child). We try to focus heavily on the fact that the girls are so big now, and we travel a lot. We have been on long journeys across the world and we go on skiing holidays. It’s so easy with these two big girls, but I still feel like I’m missing one.*^16^ (Denmark)

Weighing up the risks of pursuing subsequent pregnancies, many women felt forced to confront the possibility of dying and leaving their families. This heavily influenced their choices in many cases.

*The thing that [my husband’s name] and I have talked about is him, feeling like he’s got some concerns. [My husband says,] “What if it happens again and its worse this time, and we lose you? Then it’s me with one or two babies and I don’t have you, we don’t have you.” It’s better that it’s just the three of us then. I think he wants to really weigh the risk when we actually get to the point of really trying to get pregnant again.*^11^ (USA)

*I was told right from the beginning not to have any more children, which upsets me, but I could not go through that again or put my family through that again, maybe even leave my daughter and another child without a mother.*^18^ (OSG)

*If I want to get pregnant, I have to stop [heart medication] for at least half a year. Quitting the medication is also risky for me, but I really want to have a second child. I have talked to the doctor three times, and the nurse thinks I am too anxious ….*^17^ (Taiwan)

*I have a really hard time with it to this day. I love kids. My husband and I are both pastors. We always envisioned having a large family. At the end of the day I have to think, this is what gets me through today, it’s just that, you know, if I delivered a baby and I wasn’t here to be with my family anymore, that’s not worth it. And that’s sort of the end-all decision when I get those moments when I really want another child.*^11^ (USA)

Many women worried about the impact of not having any more children on their partner and existing children, which exacerbated their own sense of disappointment.

*For the first year and a half that was a struggle. Still I tried to be thankful for what I have; for the new life I have; for the baby I have. But I think it’s just a mother’s nature, you know; it’s just that longing. I felt bad for my son, who like, he can never have a brother or sister. I felt guilty, you know, for not giving my husband another child.*^11^ (USA)

*Me and [husband’s name] did not set a time frame for when we were going to have children. I finished college, we both worked on our careers. I wanted to start my career and do everything that married couples do. It took seven years. We planned on having two children … I waited to get pregnant until we were ready. I did everything by the book …. I feel guilty that I cannot give my mom any more grandchildren. I can’t give my husband any more children.*^11^ (USA)

In some cases, the desire for motherhood took precedence over medical advice or concern for their own health, with some women reporting that they were planning go ahead with future pregnancies. In other cases, women talked of the processes, including focusing on existing children, that ultimately helped them find acceptance of their situation.

*I cannot have any more children; it was difficult to accept since I had already had several miscarriages. I found that concentrating on my living children has helped me to get over this. I wish you luck. There is always hope. The anger at not being able to have any more children will pass.*^21^ (OSG)

## Comment

### Main findings

**Figure 2:**
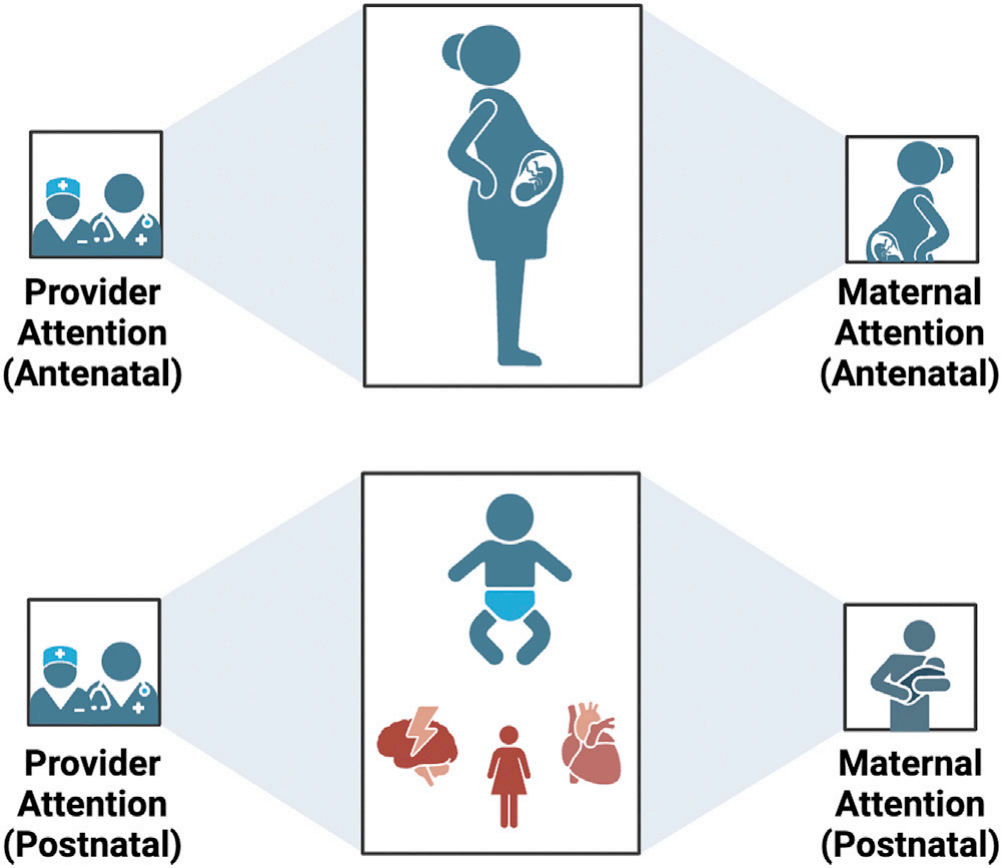
Watershed at delivery Competing demands (ie, infant, cardiac recovery) divert the attention placed on the mother’s well-being, both by herself and by her care team.

We found that women who had experienced pregnancy with a cardiac condition often had significant unmet mental and emotional well-being needs throughout the year after birth. Following delivery, we identified competing demands, including the needs of the infant and focus on cardiac recovery, that detracted attention from the mother’s well-being (Figure 2). Some women saw themselves first as a mother and found the intrusion of their cardiac care in this transition frustrating. Others felt that the focus on their infant took away clinical care from their personal health at a critical juncture in their cardiac health. Transition to motherhood often involved an important shift in mindset away from cardiac health, which was not always understood or acknowledged by care providers. Women’s experiences of new motherhood often differed drastically from their expectations, compounded by added stress and restrictions related to their cardiac condition. Emotional recovery after pregnancy complicated by a cardiac condition was often a long process lasting up to and beyond a year after birth. Women’s ability to process their experience was heavily influenced by support offered by their family and community, eg, via OSGs. Many women were actively considering whether they would be able to have another pregnancy, and for some, this was an additional source of anxiety or sadness.

Kiley. Postnatal experiences of women with cardiac conditions. AJOG Glob Rep 2025.

### Comparison with existing literature

We propose a chronological framework (Figure 1) contextualizing our findings. This framework describes the dispersal of attention at birth from the care team and within the woman’s own focus, followed by her continued transition into motherhood and physical/mindset shifts and eventual planning for the future. Women’s experiences in the postnatal period are not well-studied or understood, but our findings draw upon previous work describing the antenatal experiences of pregnant women with cardiac conditions.^8^^,^^9^

Even in uncomplicated pregnancies, postpartum recovery may be a challenging process, including struggles with mental well-being, sleep deprivation, and changing sense of self.^22^, ^23^, ^24^ Postnatal care is often overlooked and under-resourced, with known challenges in responding to individual needs.^25^ The transition to motherhood is known to be a period of profound neurocognitive development involving important hormonal and neurobiological shifts.^26^ Postpartum recovery involves an increase in oxytocin and an inflammatory stress response involving endocrine and immune mediators such as cortisol and inflammatory cytokines, while the GABAergic system plays a role in psychosocial alterations, including postpartum depression.^22^^,^^24^^,^^27^ These changes support the strong bonds that women form with their children but may also contribute to the struggles many women with cardiac conditions describe with regaining their well-being.^23^^,^^27^

Notably, women with new-onset vs pre-existing cardiac conditions had similar experiences. The vulnerabilities and challenges shared by both patient groups stemmed largely from shared difficulties in navigating postpartum recovery due to systemic healthcare issues. While women with pre-existing conditions would theoretically have had time to prepare for pregnancy, this preparation was frequently undermined by a lack of integrated care addressing their complex needs as both cardiac patients and new mothers. Our findings suggest that providing nuanced and empathetic care is a critical aspect of supporting postpartum recovery and reducing trauma associated with the cardio-obstetric patient experience.

### Strengths and limitations

An important strength of our work is that it provides insight into the experiences of an understudied, growing patient group who are at high risk of severe complications, including rising rates of maternal death.^2^^,^^28^^,^^29^ Understanding the impact of cardiac conditions after birth is the first step toward identifying effective methods of improving care. We included experiences of women across a range of countries, encompassing four continents. However, there were no available studies investigating the postnatal experiences of women with cardiac conditions in low- and middle-income countries. Further research is urgently needed to understand the implications of cardiac health on postpartum recovery worldwide, especially as patient experiences remain understudied in recent years despite the growth of cardio-obstetrics as a distinct field within maternal health. Although a wide range of cardiac conditions was represented, a large portion of the study participants were diagnosed with PPCM, emphasizing a need for future work investigating the postnatal experiences of patients with other cardiac diagnoses.

## Conclusions and implications

We highlight the profound emotional and psychosocial impact of cardiac conditions during the postnatal period. The majority of maternal deaths from cardiac disease occur in the postnatal period, and it should thus be an important focus for improving care pathways. Our findings suggest that care in the early postnatal phase needs to be carefully planned, given the difficulties of balancing the multifaceted needs of the mother, infant, and cardiac condition. Planned postnatal debriefing appointments with providers, including full discussions regarding the risks of future pregnancies, could help to address the conflicting emotions experienced by many women. Cardio-obstetric care should include supporting the transition into motherhood and providing sensitive communication to help women process their experiences and understand their long-term cardiac prognosis.

## CRediT authorship contribution statement

**Jasmine X. Kiley:** Writing – review & editing, Writing – original draft, Visualization, Project administration, Methodology, Investigation, Formal analysis, Data curation, Conceptualization. **Annabelle Corlett:** Writing – review & editing, Validation, Investigation. **Emma Mitchell-Sparke:** Writing – review & editing, Validation, Methodology. **Brittany Jasper:** Writing – review & editing, Validation, Methodology, Investigation. **Tabitha Wishlade:** Writing – review & editing, Methodology, Data curation. **Catriona Bhagra:** Writing – review & editing, Supervision, Conceptualization. **Sara Wetzler:** Writing – review & editing, Data curation. **Catherine E. Aiken:** Writing – review & editing, Writing – original draft, Validation, Supervision, Methodology, Investigation, Formal analysis, Conceptualization.

## Appendix Supplementary materials

Image, application 1

Image, application 2
