# Supplementary material for: Postnatal experiences of women with cardiac conditions: a systematic review and meta-synthesis
Source: AJOG Glob Rep. 2025 Sep 1;5(4):100564. doi: 10.1016/j.xagr.2025.100564 (PMC12509772; doi:10.1016/j.xagr.2025.100564)
Supplement: Supplementary file 2 [file mmc2.pdf]

PRISMA 2009 Flow Diagram

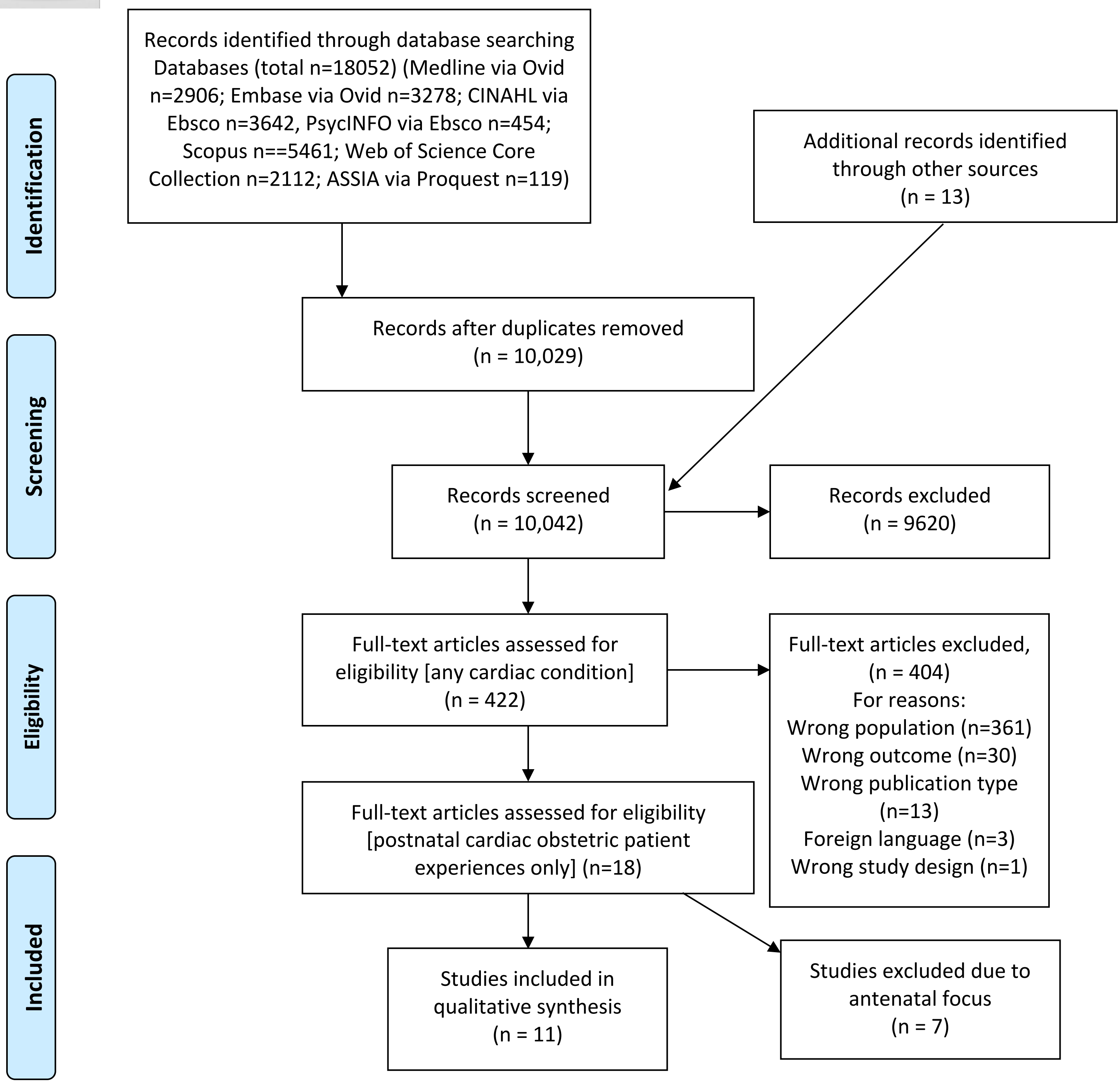

From: Moher D, Liberati A, Tetzlaff J, Altman DG, The PRISMA Group (2009). *P*referred *R*eporting *I*tems for *S*ystematic Reviews and *M*eta-*A*nalyses: The PRISMA Statement. PLoS Med 6(6): e1000097. doi:10.1371/journal.pmed1000097

For more information, visit www.prisma-statement.org.
